# Single-Cell-Precision Microplasma-Induced Cancer Cell Apoptosis

**DOI:** 10.1371/journal.pone.0101299

**Published:** 2014-06-27

**Authors:** Xiao Tan, Shasha Zhao, Qian Lei, Xinpei Lu, Guangyuan He, Kostya Ostrikov

**Affiliations:** 1 State Key Laboratory of Advanced Electromagnetic Engineering and Technology, Huazhong University of Science and Technology, Wuhan, Hubei, P. R. China; 2 The Genetic Engineering International Cooperation Base of Chinese Ministry of Science and Technology, The Key Laboratory of Molecular Biophysics of Chinese Ministry of Education, College of Life Science and Technology, Huazhong University of Science & Technology (HUST), Wuhan, P. R. China; 3 CSIRO Materials Science & Engineering, Lindfield, New South Wales, Australia; 4 School of Chemistry, Physics and Mechanical Engineering, Queensland University of Technology, Brisbane, Queensland, Australia; University Paul Sabatier, France

## Abstract

The issue of single-cell control has recently attracted enormous interest. However, in spite of the presently achievable intracellular-level physiological probing through bio-photonics, nano-probe-based, and some other techniques, the issue of inducing selective, single-cell-precision apoptosis, without affecting neighbouring cells remains essentially open. Here we resolve this issue and report on the effective single-cell-precision cancer cell treatment using the reactive chemistry of the localized corona-type plasma discharge around a needle-like electrode with the spot size ∼1 µm. When the electrode is positioned with the micrometer precision against a selected cell, a focused and highly-localized micro-plasma discharge induces apoptosis in the selected individual HepG2 and HeLa cancer cells only, without affecting any surrounding cells, even in small cell clusters. This is confirmed by the real-time monitoring of the morphological and structural changes at the cellular and cell nucleus levels after the plasma exposure.

## Introduction

The ability to isolate, manipulate, confine, interrogate and control living cells with the single-cell precision is rapidly becoming an issue of paramount importance because of the recently discovered generic phenomenon of cell-to-cell variability in their responses to external stimuli [1]–[3]. Apoptosis is one of the most important and widely studied cellular responses because of its relevance to the formation and operation of tissues and organisms at all stages of life, origin and development of diseases, as well as responses of cells to chemical therapies [4].

Given a very large number of physiological, biochemical, electrochemical and other factors that affect cellular responses, a large number of approaches are pursued [5]–[12]. These single-cell-level approaches include microelectrochemistry [5], endoscopy and interrogation based on one-dimensional nanostructures [6], [10], active and addressable microwell/microelectrode arrays [7], [9], cell manipulation, patterning, agitation, and stimulation for customized, high-precision tissue engineering [8], [11], [12], and several others. In spite of the presently achievable intracellular-level physiological probing through bio-photonics and nano-probe-based techniques, the issue of inducing selective, single-cell-precision apoptosis, without affecting neighbouring cells remains essentially open.

Recently, atmospheric-pressure gas plasmas have emerged as effective tools to induce various physiological responses in living cells and tissues including high apoptotic selectivity between malignant cancer and normal tissue cells [13]–[15]. Reduction of the plasma treatment spot sizes to micrometer dimensions has recently enabled applications of the plasma jet and corona-type discharge-induced reactive chemistry in single-cell-level treatment and highly-localized nanoparticle synthesis [16]–[20].

Even though the spot sizes of the plasma jets can be as small as 15 µm [17], which is comparable or even smaller than typical cell sizes, selective control with single-cell precision has not been demonstrated. Indeed, recent advances in the single-cell-level treatment made it possible to simultaneously expose a quite large number of isolated single cells to the plasma jet sustained in a helium flow through a thin optical fiber [16], [17]. This exposure produces a cocktail of relatively long-living chemically-active (e.g., reactive oxygen/nitrogen species, ROS/RNS) species that interact with the cells [13].

However, in the absence of precise micromanipulation and positioning of the plasma jet spot, the plasma-generated electrons and ROS/RNS species are distributed randomly in the volume of the cell culture medium and affect at least several cells that come in contact with the plasma-generated species. This conclusion is consistent with the results of statistical analysis of cell responses that suggest that a large number of cells (a significant fraction of a typical number of ∼3×10^4^ cells/well) may be affected even after a short (e.g., ∼10 s) plasma exposure and develop apoptotic responses within 24 hours after the treatment [16], [17]. Moreover, the directed He gas flow and fast propagating plasma bullets in the plasma jet may disturb the culture medium, move the cells or even cause their dehydration. This is why the issue of the plasma-enabled cell control with the single-cell precision remains essentially open.

Here we resolve this issue and report on the effective single-cell-precision cancer cell treatment using the localized corona-type plasma discharge around a needle-like electrode with the spot size ∼1 µm. When the electrode is positioned at a certain distance against a selected cell, a focused and highly-localized plasma discharge induces apoptosis in the selected individual HepG2 and HeLa cancer cells only, without affecting any surrounding cells, including small clusters of cells. This is confirmed by the real-time monitoring of the morphological and structural changes at the cellular and cell nucleus levels after the plasma exposure. Moreover, the plasma discharge is powered by a 12 V battery and is generated without any external gas flow or power supply.

## Materials and Methods

### Micro-plasma treatment

**Figure 1:**
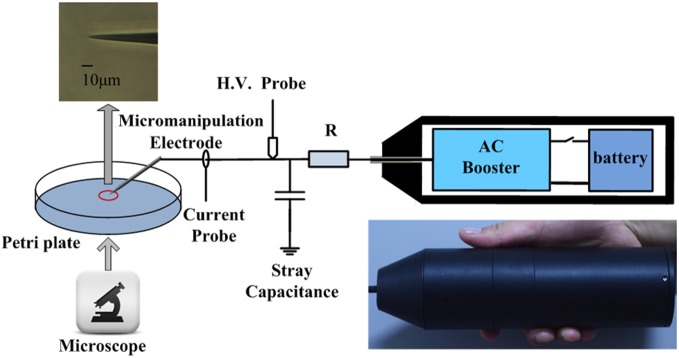
Schematic of the microplasma jet setup and a sketch of the biomedical treatment; the micro-manipulated tip has a diameter of ∼1 µm.

An electrophysiological micro- manipulator (CFT-8000D, Jiangsu, Ruiqi Co., Ltd) is used to control the electrode tip position with the precision of a few tens of nanometers (as shown in Figure 1), which makes it possible to potentially agitate selected areas (e.g., specific receptors or organelles) on the cell surface. The output of the power supply is connected to the electrode through a ballast resistor R of 10 MΩ used to limit the discharge current. This microplasma is driven by a custom-made AC power supply driven by 12 volt battery rather than any external generator or wall power. The whole weight of the microplasma device, including the power supply, is less than 200 g. The output of the AC booster can reach 5 kV with a frequency of 25 kHz. Micro-manipulatable tungsten is used as an electrode. The tip radius of the tungsten electrode is less than 0.5 µm with a taper of 13° taper angle. The diameter of tungsten probe tip is smaller than conventional cells (tens of micrometers). The gas temperature of the microplasma is close to room temperature, which suggests that the cellular response is not due to the thermal shock. A single adherent cell was selected and treated by the microplasma. The plasma exposure lasted only ∼10–15 s and the effects of the treatment were studied immediately or after several hours afterwards.

### Cell culture

Human hepatoma cancer cell line (HepG2), human cervical cancer cell line (HeLa) and normal liver cell line (L-02) were purchased from China Center for Type Culture Collection (CCTCC, Wuhan, China). The cells were cultured in high-glucose Dulbecco’s modified Eagle’s medium (DMEM, Hyclone, Logan, UT) supplemented with 10% (v/v) heat-inactivated fetal bovine serum (FBS, Hyclone) in an incubator containing a humidified atmosphere of 5% CO_2_ at 37°C. After attaining confluence, the cells were detached with 0.25% trypsin (Hyclone), seeded onto 35 mm cell culture dish (Corning, New York, USA) at the density of 2×10^4^ cells and incubated overnight to allow cell attachment.

### Real-time monitoring of morphological changes

To assess the apoptotic effect of the microplasma exposure on the treated single cell, real-time morphological observations were performed with an inverted phase contrast light microscope (XD-202, Nanjing Jiangnan Novel Optics Co., Ltd). Real-time observations of cell morphology changes were carried out and photographed. The surrounding cells were used as controls.

### Real-time fluorescence imaging of cell membrane changes

The microplasma-treated cells were stained with Annexin V-FITC following the manufacturer’s instructions (Beyotime, Jiangsu, China). Briefly, culture medium was removed and washed once with PBS. Then 500 µL binding buffer with 5 µL Annexin V-FITC was added to cells, and cultured at room temperature for 10 min in the dark. Without being washed with any liquid after reaction with the fluorochrome, a single cell was chosen and treated with the microplasma (15 s). A sufficient amount of Annexin V-FITC was left in the medium to allow binding with the translocated PS during the ongoing apoptosis process and real-time imaging process was carried out under dark conditions on a fluorescence microscope (Olympus TH4-200, Olympus Optical Co Ltd, Tokyo, Japan) immediately after the microplasma treatment. In our study, white and fluorescence images were taken immediately after Annexin V-FITC labeling procedure to record the initial conditions and the cell locations. The apoptosis onset could be monitored when the fluorescence intensity exceeded the detection threshold.

### Real-time fluorescence imaging of nucleus changes

To analyze the morphological signs of apoptotic nuclei, Hoechst 33342 staining was performed. Briefly, live HepG2 and HeLa cells were incubated with Hoechst 33342 (Beyotime, Jiangsu, China) at room temperature for 30 min. After being washed 3 times with culture medium, a single Hoechst 33342-stained cell was chosen and directly treated with the microplasma. Real-time monitoring of nucleus changes was performed using a Nikon fluorescence microscope.

## Results and Discussion

### Single-cell-precision plasma treatment

**Figure 2:**
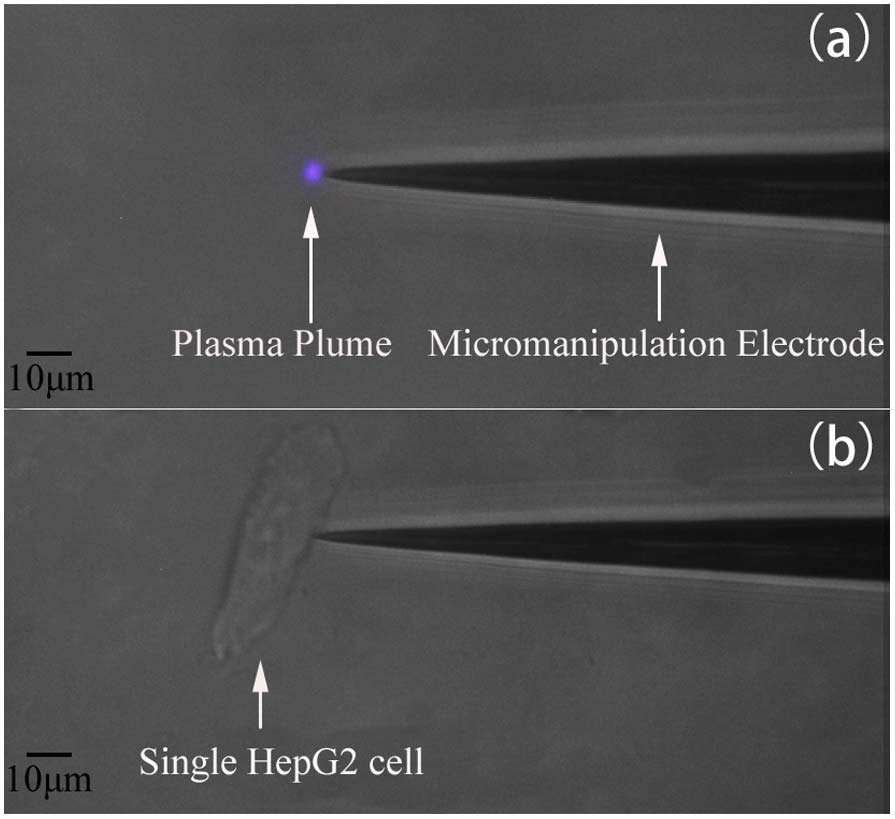
Microphotographs of (a) microplasma plume at the electrode tip; (b) single-cell-precision microplasma treatment.

Figure 2 shows the single-cell-precision microplasma treatment of a HepG2 cell. As seen in Figure 2(a), the plasma plume generated around the powered microelectrode with the tip diameter of ∼1 µm is very small (∼2 µm) and is thus suitable for the precise treatment of single cells with comparable or larger sizes. When the electrode tip approaches the cell, the cell membrane serves as a floating counter-electrode while the plasma is brought in direct contact with its surface, as seen in Figure 2(b).

**Figure 3:**
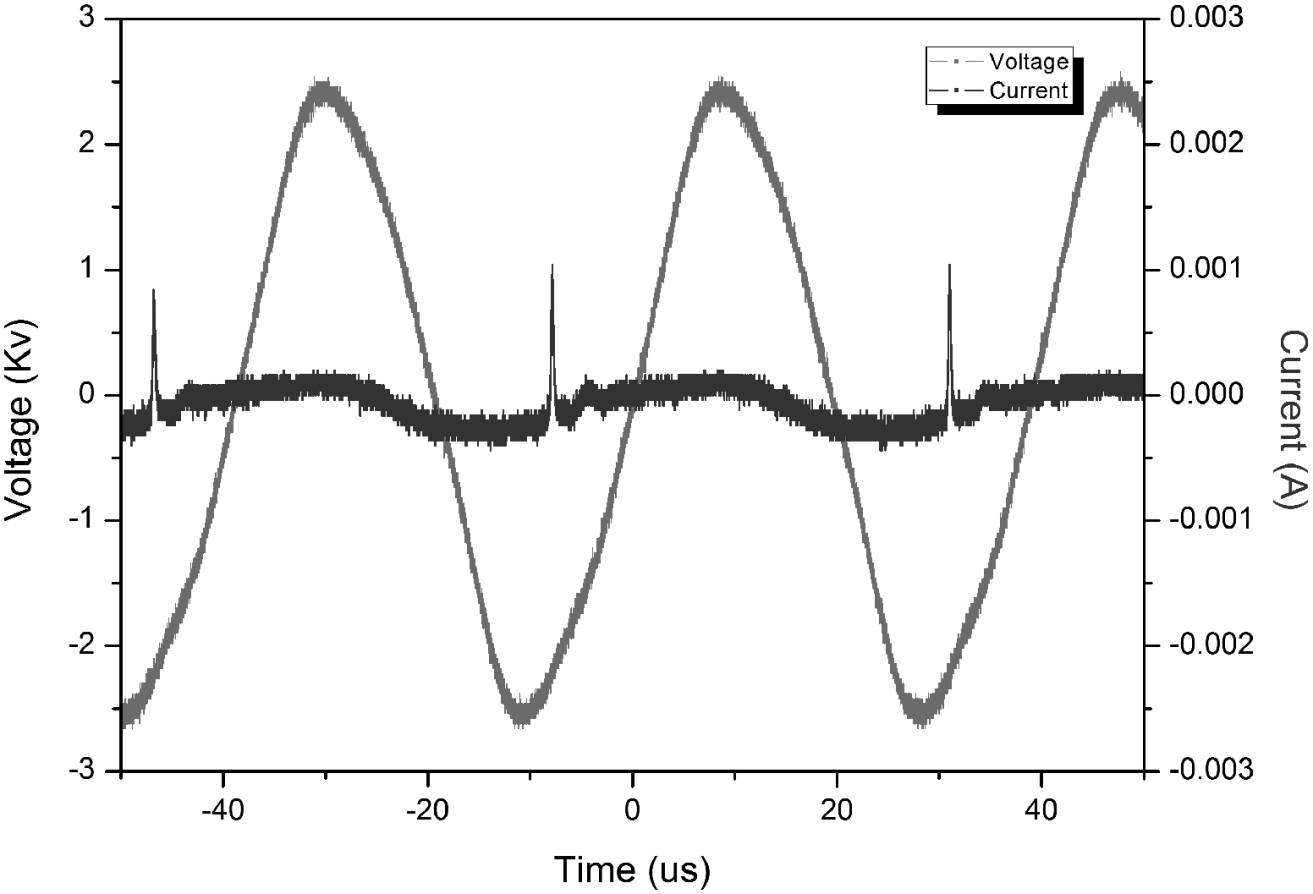
Current-voltage waveforms of the plasma discharge.

The discharge voltage and current were measured by a P6015 Tektronix HV probe and a TCP202 Tektronix current probe, respectively. They are recorded by a Tektronix DPO7104 wideband digital oscilloscope and shown in Figure 3. From Figure 3 one can clearly see that the discharge actually appears periodically with a pulse repetition rate of approximately 25 kHz. The discharge current waveform shows that the discharge occurs only once in one voltage period during the voltage rise phase. The discharge current has a full-width at half-maximum of about 270 ns and a peak value of about 1 mA. The average power delivered to the plasma is about 4 mW. The gas temperature of the microplasma is close to room temperature, and the plasma can be used directly for human skin or even internal organ treatments, without any unwanted thermal or electric shock effects.

### Cell-level morphological changes

**Figure 4:**
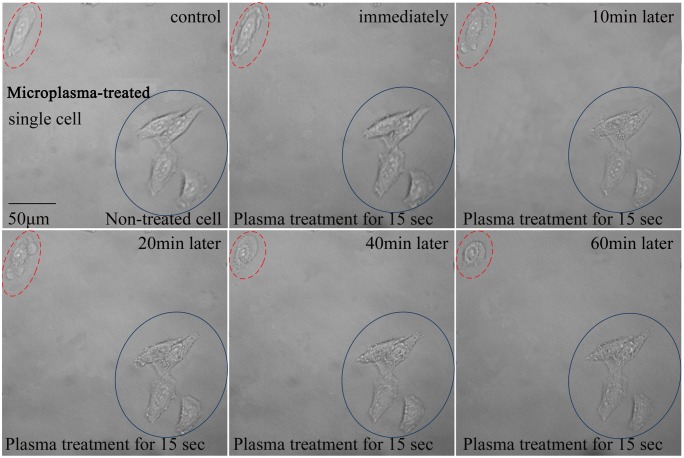
Real-time monitoring of morphological changes at the single-cell level in HepG2 cell. A single adherent HepG2 cell was selected and treated by the microplasma for 15(top left corner). The cells labeled by the blue full line are untreated control cells (bottom right corner).

To study the effect of microplasmas on the treated single HepG2 cell, morphological changes have been studied. These changes include cell contraction, membrane blebbing, chromatin condensation, DNA fragmentation, etc. and are indicative of apoptosis [21]. As seen in Figure 4, before the microplasma exposure, all the cells were healthy and spindle-shaped with clear contours. Immediately after the contact with the microplasma, the affected single cell started shrinking and gradually changed their normal shape into a more collapsed roundish shape which is quite common to apoptotic bodies.

One can see that membrane blebs appear after 20 min following the 15 s of the plasma treatment. A cell membrane deformation is also clearly seen. On the other hand, the non-treated control cells were unaffected and healthy during the entire incubation period. These distinctive morphological changes are the simplest indicators of the cell death progression, yet are not sufficient to define the apoptotic nature of the response. This is why cell membrane- and nucleus-specific fluorescent staining tests were carried out.

### Membrane-level changes

**Figure 5:**
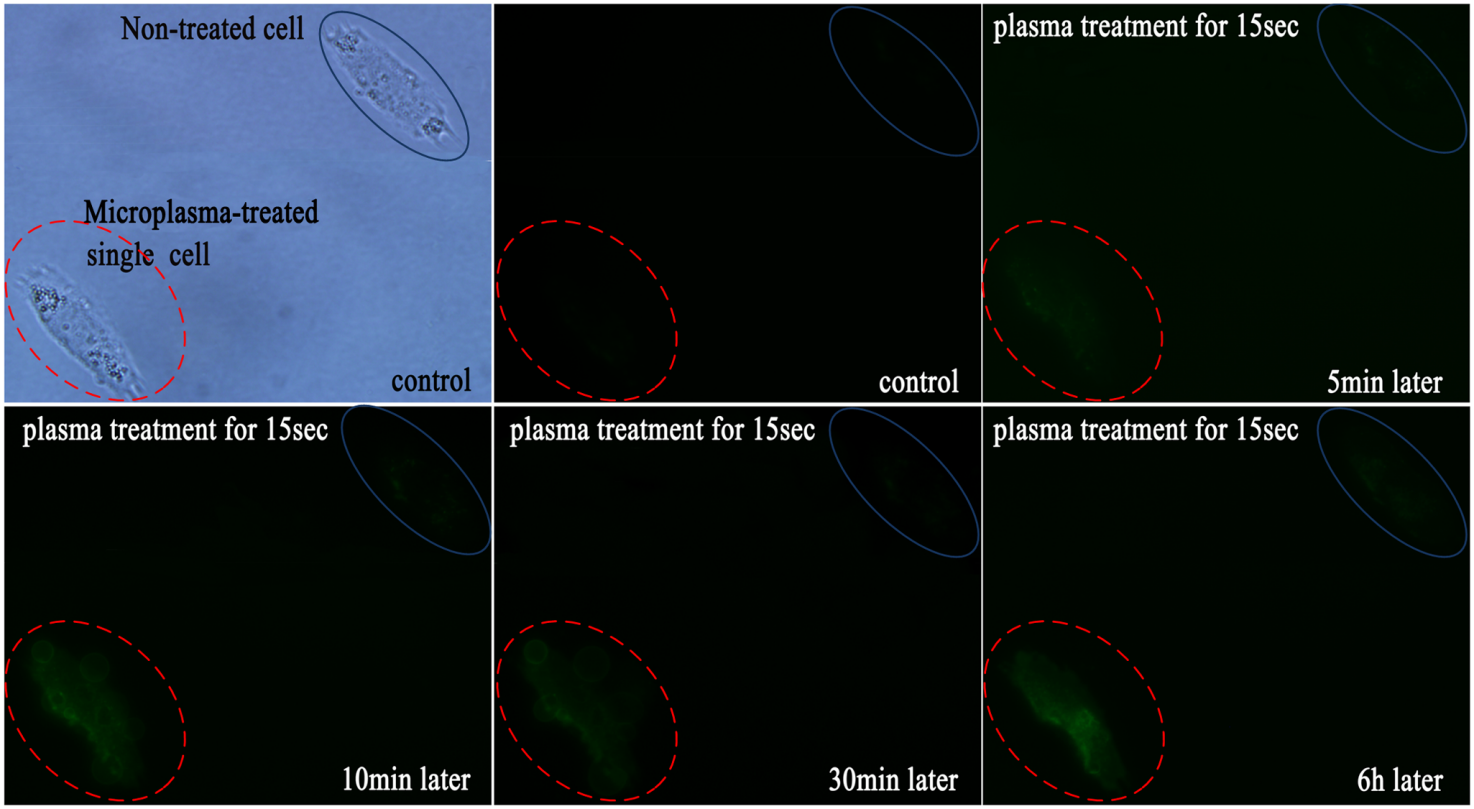
Real-time monitoring of the apoptotic membrane changes of the single HepG2 cell treated with the microplasma for 15 s. Annexin-V fluorescent staining was performed to visualize these changes. The cell labeling is the same as in Figure 4.

To validate the effect of the microplasma, real-time imaging using green fluorescent label Annexin V-FITC was performed to visualize the HepG2 cell membrane changes (Figure 5). Annexin-V has strong affinity for phosphatidylserine (PS), which is a membrane phospholipid that normally expressed only on the inner surface of the cell membrane. As the apoptosis response progresses, PS is rapidly translocated to the outer membrane surface where it is available for Annexin-V binding. The PS translocation in the membrane precedes any change in the nucleus and is regarded as one of the earliest hallmarks of cellular apoptosis [22], [23]. Thus, through binding to Annexin V, apoptotic and non-apoptotic cells can be easily distinguished visually from the fluorescence.

In the control samples, we could not observe any binding of Annexin V-FITC in HepG2 cells before the microplasma treatment. Binding of Annexin V-FITC to the plasma-treated cell became detectable 5 min after the treatment and gradually increased thereafter. However, for the non-treated cells, no fluorescence was detected even six hours later. It is noteworthy that the membrane blebbing was observed 10 min after the end of the plasma exposure. The cell became brighter and larger with a clearly fluorescent boundary upon further incubation. Membrane blebbing is another morphological change typical of apoptosis, thus this observation confirms the occurrence of apoptosis only in the microplasma-treated cell [24].

### Nucleus-level changes

**Figure 6:**
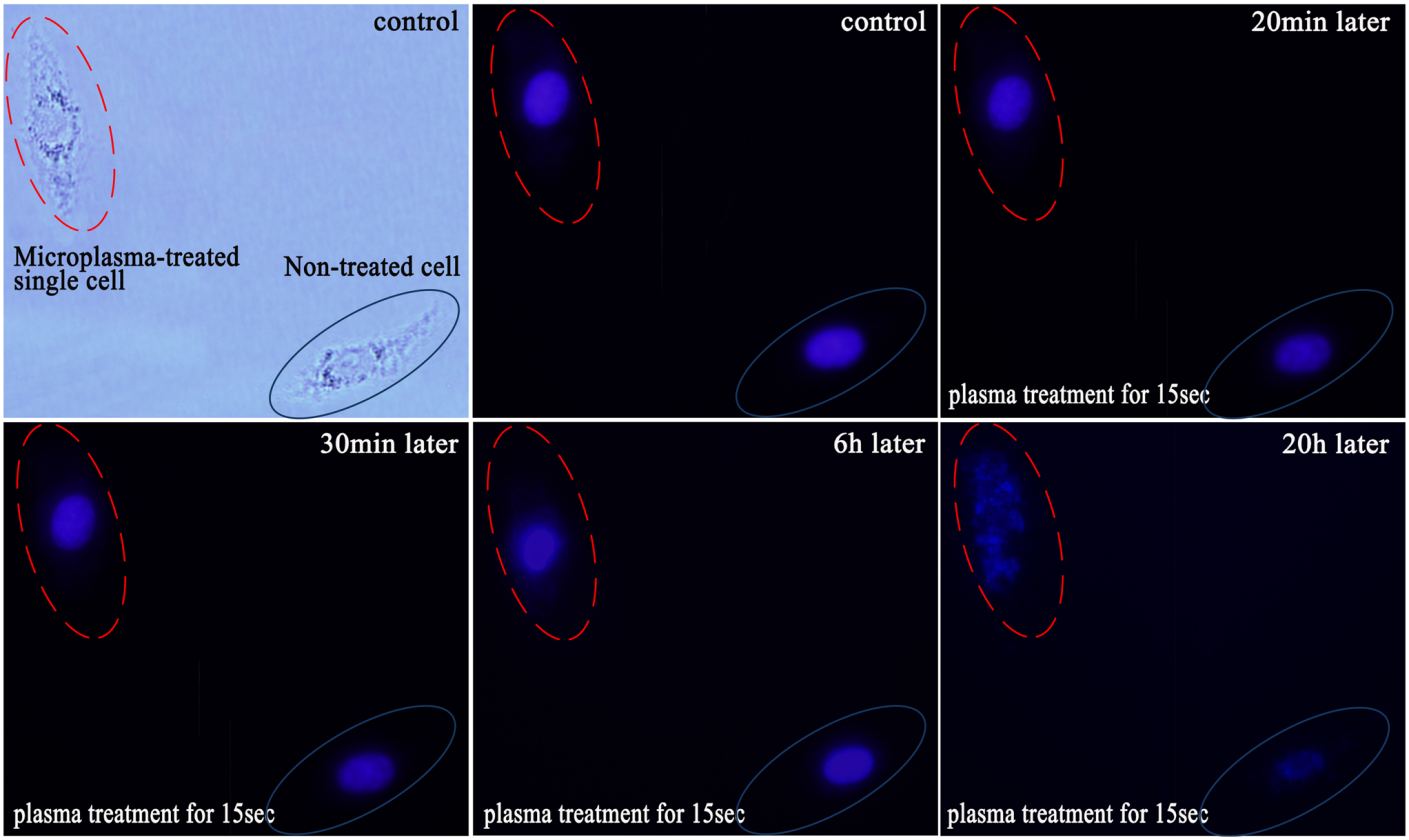
Real-time monitoring of nucleus changes of the single HepG2 cell after 15 s of the plasma treatment. The cell labeling is the same as in Figure 4.

Nucleus changes, which occurred relatively late in the process of apoptosis, were imaged with DNA staining by Hoechst 33342 dye. Similar to the membrane staining, white and fluorescence images were taken immediately after the Hoechst 33342 labeling to record the initial conditions and the cell locations (first two images in Figure 6). The cell in the top left corner was treated by the plasma for 15 s while the cell in the bottom right corner was the control cell. At the beginning, both treated and un-treated cells were stained blue with the same brightness. Since nucleus changes occur relatively late in the process of apoptosis, no obvious difference can be seen even at 20 min after the microplasma treatment. However, 30 min after the plasma exposure, nucleus of the treated single cell became brighter compared to the non-treated cell.

The observed difference in the fluorescence intensity may be due to, on the one hand, the dysfunction of P-glycoprotein, a membrane transporter which could extract the Hoechst 33342 in the cell. However, P-glycoprotein pump function is usually impaired and could not effectively transport Hoechst 33342 out of the apoptotic cell. This causes accumulation and hence stronger emission of Hoechst 33342 from the apoptotic cell [25], [26]. On the other hand, nucleus began to condense, causing relatively stronger fluorescence. 6 hour later, nucleus condensation was clearly seen in the treated single cell. Thereafter, the nuclear membrane was clearly disrupted, accompanied by diffused DNA fragments. On the country, non-treated cells retained a normal nuclear morphology.

### Possible effects of reactive species

**Figure 7:**
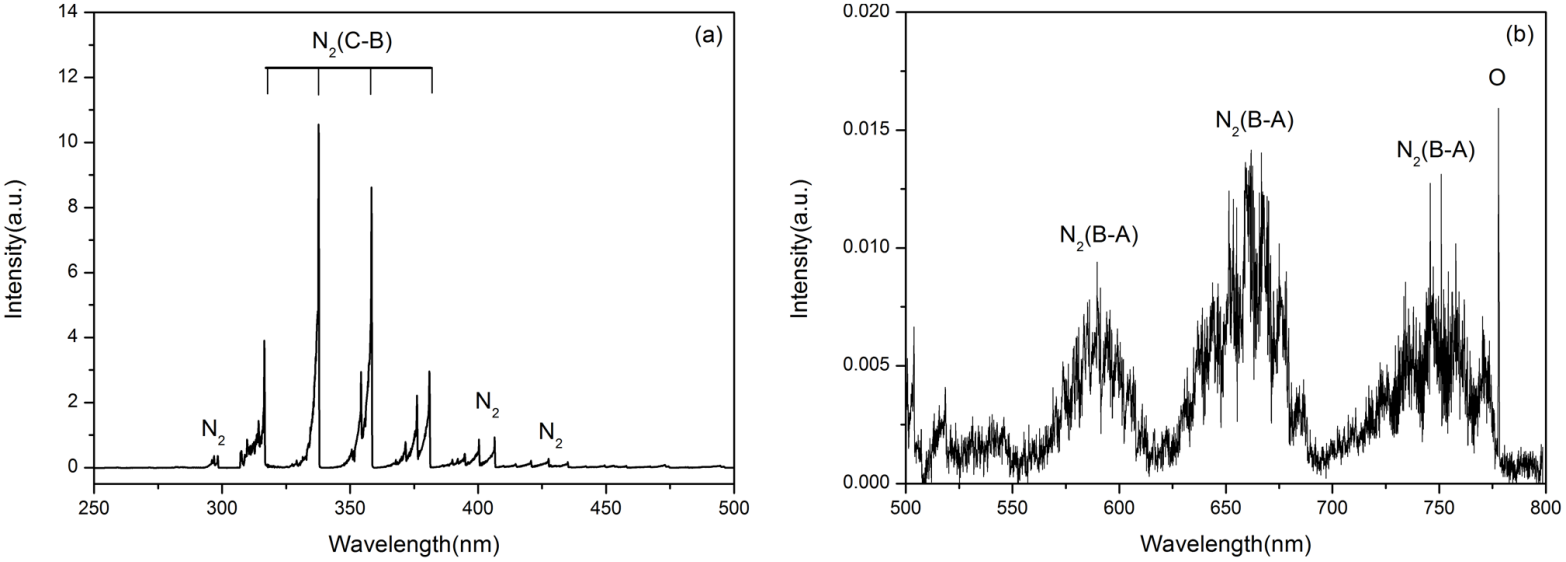
Optical emission spectra of the plasma: (a) 250–500 nm and (b) 500–800 nm.

To characterize chemically active species in the microplasma, optical emission spectroscopy (OES) is used, which allows the analysis of the radiation emitted by the atoms, ions, molecules, and radicals. Hence, a half-meter spectrometer (Princeton Instruments Acton SpectraHub 2500i; spectral resolution: 2 nm, grating: 1200 g mm^−1^, slit width: 150 um) is used to measure the optical emission of the microplasma plume. Figure 7 shows the optical emission spectra (OES) of the microplasma plume in the 250–800 nm spectral range. As can be seen, the optical emission spectra are dominated by the excited nitrogen and oxygen species.

The ROS play a crucial role in cancer cell death, and can induce apoptosis by affecting the DNA, as well as lipids and proteins that are involved in intracellular signaling cascades [27]. Excessive production of ROS may either directly damage the cellular structure to cause cell necrosis or indirectly affect normal cellular signaling pathways and gene regulation to induce apoptosis [28]. The reactive nitrogen species may also affect the cell-inactivation process [13]. In particular, NO radicals can cause apoptosis, necrosis or, alternatively, protect the cells from death, depending on the cell type, radical concentration, as well as the duration and specific areas of the exposure [29]. Numerical simulations of the active species generated in tip-sustained corona-type plasma discharges [30] and their extension towards biologically-relevant media are therefore highly warranted in the near future.

### Plasma versus electric field effects

The micro-tips in our experiments also generate significant time-variable electric fields in their vicinity. As reported previously [31], pulsed electric fields may also cause cell death, e.g., through electric pulse-induced electroporation of the cell membrane. In the close vicinity of the micro-tip used in our experiments, the magnitude of the electric field can reach tens and even hundreds of kilovolts per centimeter. However, the electric field very rapidly decreases with distance away from the tip. In our experiments the electrode tip was typically positioned approximately 150 µm away from the cell surface. Therefore, the cell membranes experienced much weaker electric fields than near the tip’s surface, with the estimated magnitude of about 1–2 orders of magnitude lower. This is why the probability of merely electric-field-induced cell death is lower in our experiments compared to the electric field effects reported previously [31].

**Figure 8:**
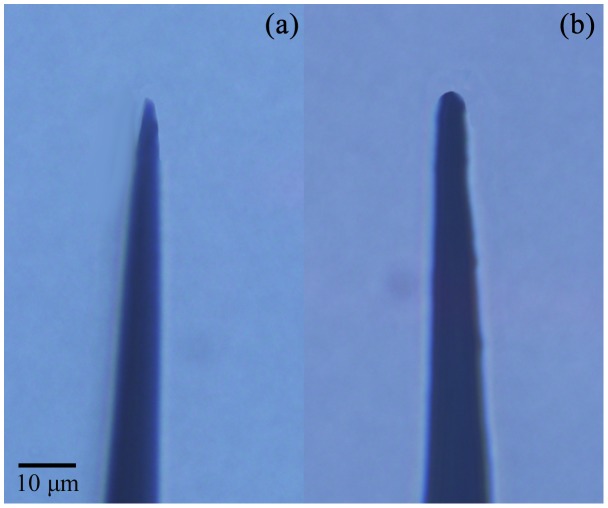
Uncoated (a) and wax-coated (b) microtips used in our experiments to elucidate the effects of a plasma versus electric field effects.

To demonstrate the primary importance of the reactive species generated by the microplasma discharge compared to the electric field-related effects, we have conducted a set of control experiments where the microtip was coated with a very thin layer of paraffin wax which prevents the plasma generation, yet not very significantly disturbs the magnitude of the electric field (at least it remains of the same order of magnitude as for the plasma-generating uncoated microtip). The uncoated and paraffin wax-coated microtips that were used in these studies are shown in Figure 8.

Importantly, the cells treated with the wax-coated microtip did not experience apoptosis, which can be interpreted that the observed apoptotic responses of the cells are indeed more related to the plasma-generated species rather than merely the electric field effects. Specifically, to elucidate the effects of the plasma exposure versus the electric field-related effects, we treated four HepG2 cells using both pristine and wax-coated microtips shown in Figures 8(a) and (b), respectively.

**Figure 9:**
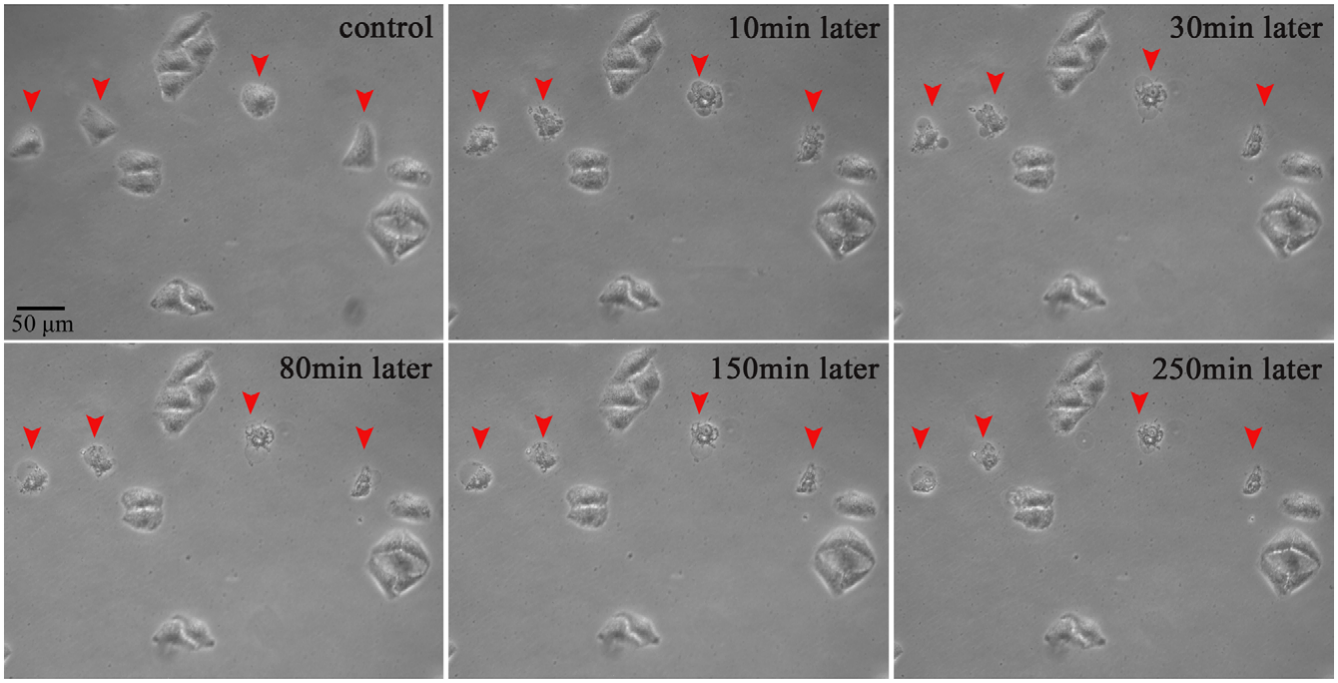
Morphological evolution of the membrance blebbing in 4 selected HepG2 cells treated with microtip in **Figure 8(a)**.

Morphological studies of the cells subjected to the plasma exposure have been carried out. As seen in Figure 9, before the microplasma exposure, all the HepG2 cells were healthy and spindle-shaped with clear contours. Four cells were selected randomly and treated with a pristine microtip (shown in Figure 8(a)) for 15 s. Membrane blebs appeared approximately 10 minutes after the exposure and their membrane deformation is also clearly seen with the prolonged incubation time. On the other hand, non-treated cells were unaffected and healthy during the entire incubation periods.

**Figure 10:**
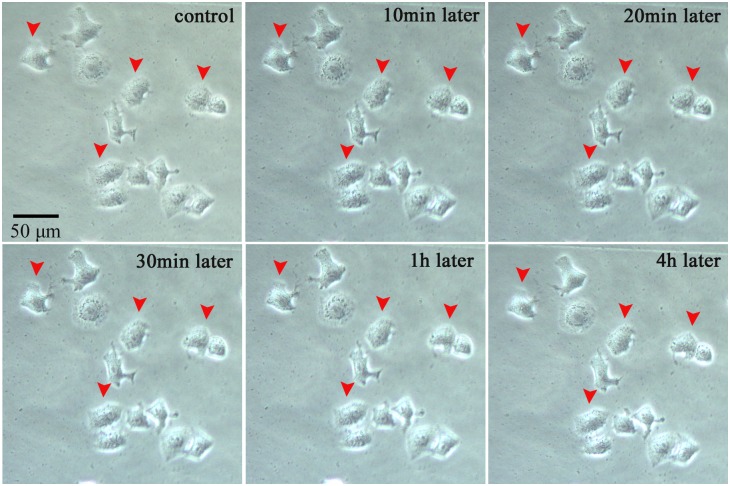
Same as in **Figure 9** for wax-coated microtip in **Figure 8(b)**.

Exactly the same treatment of the four randomly selected HepG2 cells using the wax-coated microtip shown in Figure 8(b) showed completely different results. In Figure 10, one can clearly see that the four cells are not affected significantly.

These observations clearly support our conclusion that the effects observed in our microplasma experiments are more closely related to the reactive species generation in the plasma. These effects may also be quite different from the commonly known electric field effects such as electroporation.

### Normal cells are not affected by plasma treatment

**Figure 11:**
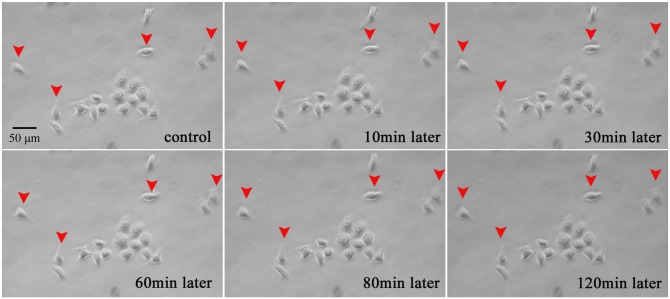
Plasma treatment of 4 selected normal liver cells does not noticeably affect them, even after 2 hours of observation.

We have also evaluated the effects of the microplasma exposure on a normal liver cell line (L-02) with the same microplasma and treatment parameters. As shown in Figure 11, the plasma treatment of the four randomly selected L-02 cells did not lead to any significant effects, even after 2 hours of observation. This result opens an opportunity for the detailed parametric and process optimization studies, which will be a subject of our future work and possibly work of other researchers.

### Effects of plasma on other cancer cells

**Figure 12:**
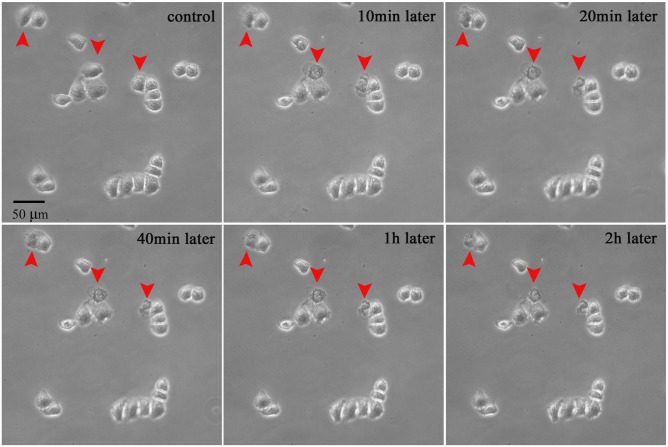
Morphological evolution of 3 selected HeLa cells treated with microplasmas.

**Figure 13:**
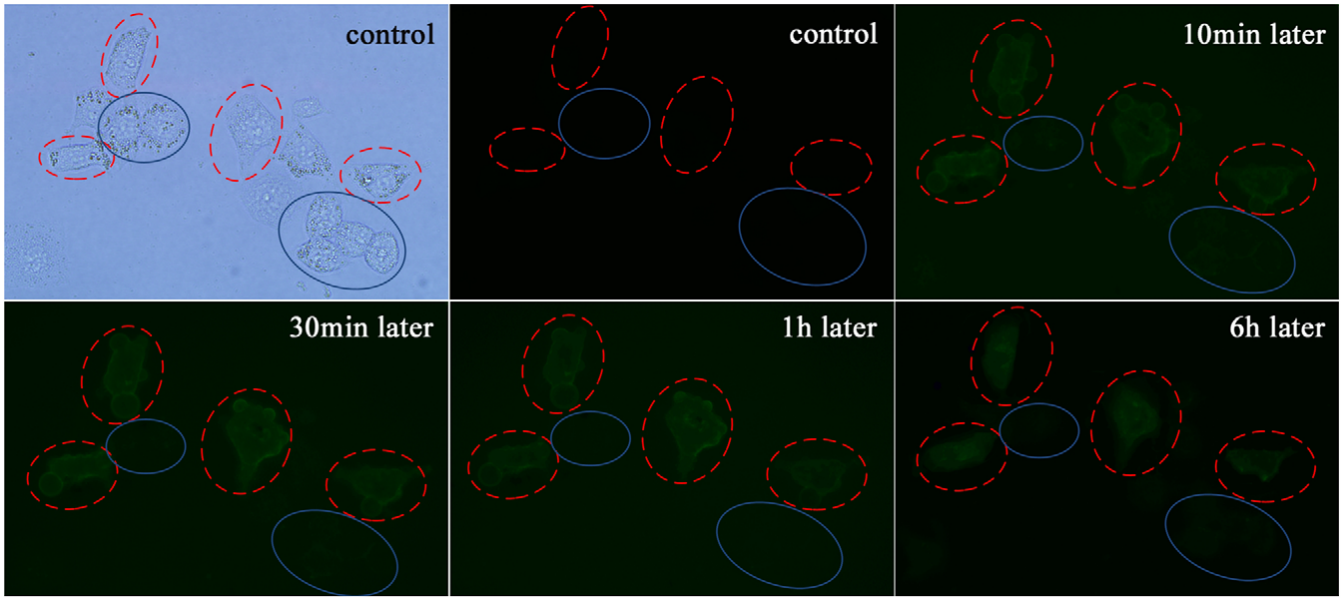
Annexin-V staining suggests that the 4 microplasma-treated HeLa cells show apoptotic response.

**Figure 14:**
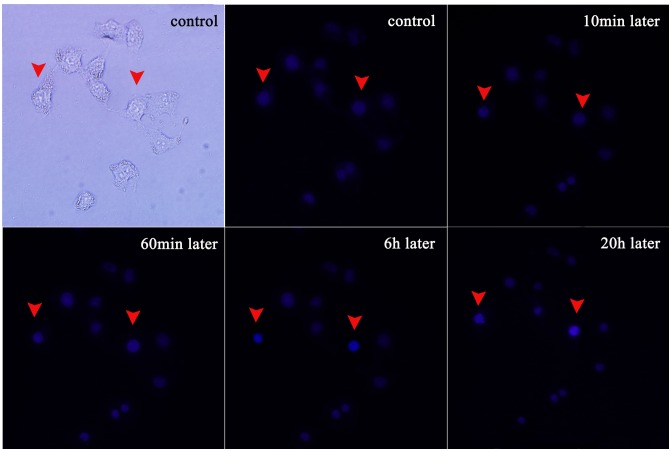
Hoechst 33342 staining further confirms nucleic changes indicative of apoptosis. The blue fluorescence from the plasma-treated cells is much stronger than from untreated cells.

Another cancer cell line (HeLa) were used to clarify the effect of the microplasma exposure. In Figures 12–14, the treated cells and untreated cells are closely contacted to form small compact cell clusters. Importantly, just the plasma-treated cells were killed, while the neighboring cells were not affected significantly. This can be clearly seen from the 2 hours-long examination of the morphological changes shown in Figure 12.

The results of Annexin V-FITC (Figure 13) and Hoechst 33342 (Figure 14) staining further confirmed these results. Just the microplasma-treated HeLa cells showed nucleus condensation and the PS translocation with Hoechst 33342 accumulation and Annexin V staining, which are the indicators of cell apoptosis. Therefore, we can conclude that the microplasma exposure is indeed sufficient to induce apoptosis selectively, without affecting neighboring cells.

### Limitations of single-cell-precision studies

These studies were limited to simple indicators of the cell apoptosis, without aiming to study the cell cycle or intracellular mechanisms compared to recent publications [32], [33]. The main reason is due to the limitations of the flow cytometry, Western blot, and q-PCR techniques to determine the apoptotic responses from individual cells. As can be seen in Figures 9–14 above, the short microplasma exposure is sufficient to induce apoptosis just in the treated cancer cells, while neighboring cells are not affected. However, the flow cytometry and Western blot methods typically require at least 10^4^ cells, and the methods are often used to study the changes of a relatively large cell population. The single-cell-precision cell apoptosis may not show changes detectable by the flow cytometry and Western blot methods. This is why we used simple Annexin V and Hoechst 33342 staining as indicators of cell apoptosis, and found that the microplasma used in this work indeed leads to apoptosis with a single-cell precision.

### Potential applications

The results of our work are important for several future biomedical applications. For example, with the advent of early-stage detection of a small number of cancerous cells it may be possible to selectively treat the malignant cells while keeping the normal cells intact. It presently remains very challenging to identify very small clusters of cancerous cells below a certain minimum number of cells. When the relevant unambiguous early detection techniques become available, the microplasma-based approach of this work may potentially make a significant impact on the cancer treatment therapies.

Other possibilities include agitation of selected areas on cell surfaces for inducing specific cellular effects such targeted reprogramming of stem cells (possibly including cancer stem cells) to achieve the desired potency and fate choice outcomes, as well as intracellular probing and agitation of the selected organelles. In these cases the effects of time-varying electric fields also need to be unambiguously included and interpreted.

## Conclusion

In summary, we have demonstrated that individual HepG2 and HeLa cancer cells can be effectively inactivated via the single-cell-precision, microplasma-induced apoptotic response, while normal L-02 liver cells remain unaffected by the same plasma exposure. The microplasma can be confined to the small (∼1 µm) volume around the tip of a needle which can be positioned in any specific area by using a micromanipulator. The power delivered to the cell is very small (a few mW) yet sufficient to induce apoptosis selectively, without affecting neighboring cells, even within small clusters of closely contacting cells. The plasma source is battery-operated and does not rely on any external power or gas supplies, which may be particularly useful in situations where external power supply is not available or device portability is an issue.

This advance is generic and applicable to different types of cancer cells. It may lead to next-generation single-cell-precision microsurgeries. Step changes are also possible in the capability of addressable microarrays towards instantaneous inactivation of the as-detected malignant cells, where the needles in the arrays may be used as both the electrophysiological probes and the electrodes for the plasma generation.
